# Whole-genome sequencing of the hemorrhagic septicemia vaccine strain in Bangladesh

**DOI:** 10.1128/mra.00625-26

**Published:** 2026-07-10

**Authors:** Parinita Basak, Md. Rezaul Karim, Rabeya Akhter Banu, Sadia Khanam, Md. Mostofa Kamal, Mohammed Abdus Samad

**Affiliations:** 1 Livestock Research Institute 560480, Dhaka, Bangladesh; 2 Animal Health Research Division, Bangladesh Livestock Research Institute 560480, Savar, Dhaka, Bangladesh; 3 Transboundary Animal Disease Research Center, Bangladesh Livestock Research Institute 560480, Savar, Dhaka, Bangladesh; DOE Joint Genome Institute, Berkeley, California, USA

**Keywords:** hemorrhagic septicemia vaccine, *Pasteurella multocida*, whole-genome sequencing

## Abstract

Hemorrhagic septicemia (HS) is an acute and economically important bacterial disease of cattle and buffalo caused by *Pasteurella multocida*. In this study, we report the whole-genome sequence of the HS vaccine strain, *P. multocida* BLRI-LRI-HS (Bangladesh Livestock Research Institute-Livestock Research Institute-hemorrhagic septicemia-vac), used for vaccine production in Bangladesh. The assembled genome was approximately 2.3 Mbp, with 40.5% GC content.

## ANNOUNCEMENT

*Pasteurella multocida*, an opportunistic pathogen, is a gram-negative, non-motile, encapsulated, non-spore-forming bacterium, occurring singly, in pairs, or occasionally as chains or filaments, belonging to the *Pasteurellaceae* family, which infects a wide variety of domestic and wild animals, including poultry and ruminants (1). In cattle and buffalo, this bacterium is recognized as a major etiological agent of bovine respiratory disease complex and hemorrhagic septicemia (HS), both of which cause substantial economic losses due to high morbidity and mortality (1, 2). *P. multocida* strains are serologically divided into five capsular serogroups (A, B, D, E, and F) based on their capsule antigens, while classification based on lipopolysaccharide somatic (O) antigens categorizes the organism into 16 distinct serotypes. Here, we present the genome sequence of *P. multocida* strain isolated from the HS vaccine, providing genomic data relevant to its sequence identity, multilocus sequence typing (MLST) type, genome composition, and genetic stability. The HS vaccine strain master seed obtained from the Bacterial Vaccine Production Unit of Livestock Research Institute (LRI), Bangladesh, was enriched overnight at 37°C in Luria-Bertani broth (Oxoid, Thermo Fisher Scientific, UK). Subsequently, a loopful of the enriched culture was streaked onto blood agar base supplemented with 5% sheep blood (Oxoid, Thermo Fisher Scientific, UK) and incubated at 37°C for 24–48 h. Genomic DNA was extracted from a single colony using the QIAamp DNA Mini Kit (Cat. No. 51304, Germany) following the manufacturer’s protocol. DNA concentration was measured using the Qubit dsDNA Quantification Assay, yielding 11.7 ng/µL. A total of 400 ng of genomic DNA was used for library preparation with the Illumina DNA Prep Library Preparation Kit (Illumina, Inc., San Diego, CA, USA). Sequencing was performed with 2 × 150 bp paired-end chemistry on Illumina NextSeq 2000. Sequencing of strain BLRI-LRI-HS-vac (Bangladesh Livestock Research Institute-Livestock Research Institute-hemorrhagic septicemia-vac) generated 1.73 M reads, with an average read length of 150 bp. FastQC v0.12.1 (3) was used to check the quality of reads, and the reads were filtered using Fastp v0.24.0 (4). The genome was assembled using SPAdes v. 4.0.0 (5) into 78 contigs at least 200 nucleotides long, and the post-quality resulting assembly was checked with QUAST v5.3.0 (6). To assess assembly accuracy, genome completeness and contamination were determined with BUSCO v5.8.0 (7), and average nucleotide identity (ANI) values were determined through FastANI v1.3 (8) to a reference (accession no. GCF_002073255.2). Genome annotation of BLRI-LRI-HS-vac was performed using the National Center for Biotechnology Information (NCBI) Prokaryotic Genome Annotation Pipeline v6.9 (9). All analyses were performed using default settings, unless otherwise specified.

The assembled genome of *P. multocida* strains comprises 2.3 Mbp spanning 78 contigs with genome coverage ~100×. The GC content was 40.5%. Assembly quality metrics included L50 values of 7, with corresponding N50 values of 105.1 kb. BUSCO analysis demonstrated 98.3% completeness with only 0.1% duplicated BUSCOs, indicating a highly complete and low-contamination genome assembly. The ANI value of 98.54% confirmed close genomic relatedness with the reference *P. multocida* genome (GCF_002073255.2). A summary of genome characteristics is provided in Table 1.

**TABLE 1:** General features of the complete genome sequence of hemorrhagic septicemia vaccine strain, *P. multocida* BLRI-LRI-HS-vac

Genomic feature	*P. multocida* BLRI-LRI-HS-vac
BioSample accession	SAMN55381577
GenBank accession	JBVFWZ000000000.1
Assembly accession	GCF_055522355.1
SRA	SRR38671233
Genome size	2.3 Mb
GC content	40.5%
Genes (total)	2,210
CDSs (total)	2,148
Genes (coding)	2,108
CDSs (with protein)	2,108
Genes (RNA)	62
rRNAs	3, 1, 1 (5S, 16S, 23S)
Complete rRNAs	2, 1, 1 (5S, 16S, 23S)
Partial rRNAs	1 (55)
tRNAs	53
ncRNAs	4
Pseudogenes (total)	40
CDSs (without protein)	40
Pseudogenes (ambiguous residues)	0 of 40
Pseudogenes (frameshifted)	11 of 40
Pseudogenes (incomplete)	21 of 40
Pseudogenes (internal stop)	11 of 40
Pseudogenes (multiple problems)	2 of 40
BUSCO completeness	98.3
BUSCO contamination	0.1
Average nucleotide identity (ANI)	98.54
N50	105.1 kb
N90	28,674
auN	120,449
L50	7
L90	24

## Data Availability

The Whole Genome Shotgun project of the hemorrhagic septicemia vaccine strain, *P. multocida* BLRI-LRI-HS-vac has been deposited in GenBank and the NCBI Sequence Read Archive (SRA) under accession SRR38671233 and BioProject accession PRJNA1424275. The version described in this paper is version JBVFWZ000000000.1.
